# Comparisons of conventional and novel anthropometric obesity indices to predict metabolic syndrome among vegetarians in Malaysia

**DOI:** 10.1038/s41598-020-78035-5

**Published:** 2020-11-30

**Authors:** Yuan Kei Ching, Yit Siew Chin, Mahenderan Appukutty, Wan Ying Gan, Yoke Mun Chan

**Affiliations:** 1 Department of Nutrition and Dietetics, Faculty of Medicine and Health Sciences, Universiti Putra Malaysia, Serdang, Selangor Malaysia; 2 Research Centre of Excellence, Nutrition and Non-Communicable Diseases, Faculty of Medicine and Health Sciences, Universiti Putra Malaysia, Serdang, Selangor Malaysia; 3 Programme of Sports Science, Faculty of Sports Science and Recreation, Universiti Teknologi MARA, Shah Alam, Selangor Malaysia

**Keywords:** Diseases, Pathogenesis, Risk factors

## Abstract

Our study aimed to compare the ability of anthropometric obesity indices to predict MetS and to determine the sex-specific optimal cut-off values for MetS among Malaysian vegetarians. Body weight, height, waist circumference (WC), blood pressure (BP), fasting venous blood sample were collected from 273 vegetarians in Selangor and Kuala Lumpur, Malaysia. The abilities of body mass index (BMI), body fat percentage (BF%), waist to height ratio (WHtR), lipid accumulation product (LAP), visceral adiposity index (VAI), a body shape index (ABSI), and body roundness index (BRI) to identify MetS were tested using receiver operating characteristic (ROC) curve analyses. MetS was defined according to the Joint Interim Statement 2009. The ROC curve analyses show that BMI, BF%, WHtR, LAP and VAI were able to discriminate MetS in both sexes. LAP was a better predictor to predict MetS, followed by WHtR for male and female vegetarians. The suggested WHtR’s optimal cut-offs and LAP’s optimal cut-offs for MetS for male and female vegetarians were 0.541, 0.532, 41.435 and 21.743, respectively. In conclusion, LAP was a better predictor to predict MetS than other anthropometric obesity indices. However, WHtR could be an alternative obesity index in large epidemiology survey due to its convenient and cost-effective characteristics.

## Introduction

Metabolic syndrome (MetS) is recognised as the leading risk factor of cardiovascular diseases (CVD) and type II diabetes mellitus (T2DM)^1^. It is characterised by the presence of at least three of the following risk factors namely abdominal obesity, elevated blood pressure (BP), elevated fasting blood glucose (FBG), elevated triglyceride (TG) and low level of high-density lipoprotein cholesterol (HDL-c)^1^. The global prevalence of MetS is estimated to affect about 20.0–25.0% of the adult population, which may vary across different populations and definitions used to define MetS^2^. Notably, MetS affected the nations in both developed and developing countries. For instance, the prevalence of MetS increased from 32.9% in 2004 to 34.7% in 2015 among the United States population^3^. The adjusted prevalence of MetS was 24.3% based on 12 cohorts’ studies in European countries^4^. In a developing country such as Indonesia, the prevalence of MetS was 21.6%^5^. Meanwhile, a systematic review conducted in the Asia Pacific regions which consists of eight countries, namely Malaysia, Singapore Philippines, Sri Lanka, South Korea, China, Taiwan, and Mongolia shown that the prevalence of MetS ranged from 11.9 to 37.1%^6^. A recent meta-analysis conducted among the Gulf Cooperation Council (GCC) such as Oman, Qatar, Kuwait, United Arab Emirates, Saudi Arabia and Bahrain found that the pooled overall prevalence of MetS was 28.0%^7^. In Malaysia, about two in five adults (42.5%) had MetS^8^.

Researchers revealed that the rising trend of MetS was attributed to the obesity epidemic^9,10^. In cognisance of the prevailing of MetS and obesity, several efforts have been made in order to identify a diagnostic tool to predict MetS at the early stage. A dual-energy x-ray absorptiometry (DXA) and computerized tomography (CT) scan are advanced measurements to assess the adiposity level in the human body^11,12^. However, DXA and CT are not applicable to be used in large scale epidemiology surveys due to its expensive and time-consuming characteristics^13^. In contrast, the anthropometric obesity index is a cost-effective tool to estimate the degree of obesity in large scale epidemiology surveys^12^. Receiver operating characteristic (ROC) curve analysis aimed to compare the performance of the diagnostic tool in predicting human diseases^14^. A specific optimal cut-off to predict certain disease could be generated from the ROC curve analysis according to the largest Youden Index (YI)^14^. In the past, the abilities of various conventional anthropometric obesity indices such as body mass index (BMI), body fat percentage (BF%), and waist to height ratio (WHtR) to predict MetS had been tested using ROC curve analyses in different populations^15–17^. Considering the differential risk of MetS between sexes, some studies also suggested sex-specific optimal cut-off in predicting the MetS and cardiovascular risk factors based on the YI^18,19^.

Although BMI is the most common method to classify obesity^20^, it was unable to distinguish the muscle mass from the fat mass^21^ as well as upper abdominal obesity from central obesity^22^. Instead of using a proxy indicator to measure obesity, application of bioimpedance analyses (BIA) is an indirect measurement of body fat. BIA had gained its popularity due to its ability to differentiate between lean mass and fat mass^23^. On the other hand, WHtR is suggested to be used to predict MetS, cardiometabolic risk factors and mortality^24–26^. However, WHtR does not distinguish between the subcutaneous fat from the visceral fat. Several studies have revealed that visceral fat had a stronger association with MetS and cardiometabolic risk factors as compared to subcutaneous fat^27,28^. The inconsistencies between the studies highlight that further researches are needed in order to have a better understanding of the discriminatory ability for each index, specifically among those on a vegetarian diet.

Lipid accumulation product (LAP) and visceral adiposity index (VAI) are novel indices to measure visceral obesity^29^. Previous studies have shown that LAP had a better correlation with MetS^30^, whereas VAI has been suggested as an indicator of adipose distribution to measure the cardiometabolic risk before it progresses into MetS^31^. Nonetheless, it is not cost-effective when using LAP and VAI to predict MetS as both of these indices were generated from the blood lipid parameters. Hence, a body shape index (ABSI) and body roundness index (BRI) were proposed as alternatives to measure visceral obesity. ABSI aims to determine abdominal obesity based on body shape without collecting the blood lipid parameters from laboratory analyses^32^. ABSI had better predictability to determine the metabolic profiles than BMI and WC in the United States^32^ and the British population^33^. Nonetheless, some researchers found that ABSI was not effective to predict hypertension^34^, insulin resistance^35^ as well as MetS^36,37^. Last but not least, past literatures have reported the potential of using BRI in the assessment of hypertension than BMI, ABSI, and WHtR^34^. However, a subsequent study showed that WHtR had a better ability to predict MetS than BRI among the Polish population^17^.

Literatures suggested that dietary patterns play a role in the pathogenesis of MetS^38,39^, whereby individual on a vegetarian diet had a lower risk of MetS than non-vegetarians^39,40^. Nonetheless, our recent study found that MetS was prevalent, with about one in four vegetarians in Malaysia had MetS^41^. Up to-date, there is no published findings to indicate the prevalence of vegetarians in Malaysia. However, the rising demand for vegetarian food products such as mock meat products^42^ as well as the increasing number of scientific publications among Malaysian and overseas vegetarians^42–46^ indicate that the vegetarian diet has an increasing popularity worldwide. Despite literatures review demonstrated that vegetarians had a lower prevalence of MetS than non-vegetarians^39,46^, the MetS issues in vegetarians should not be neglected. The increasing trend of vegetarianism and MetS issues highlights the needs to establish a suitable diagnostic tool to predict MetS among vegetarian population.

Up to-date, most of the studies did not separate the indices into the conventional anthropometric obesity indices and novel anthropometric obesity indices category^47,48^. It is impractical to use LAP and VAI in a large community survey, whereby LAP requires the input of TG^49^ and VAI requires the input of TG and HDL-c^31^. In contrast, conventional anthropometric obesity indices that can be performed without the blood parameters could be better options to predict MetS, especially in a large community project. Despite so, it is crucial to know the differences between conventional anthropometric obesity indices and novel anthropometric obesity indices to predict MetS. These comparison data provide a summary of the overall performance of these anthropometric obesity indices in predicting MetS, which provide more options for the researchers and health care professionals in clinical settings to decide which anthropometric obesity index to be used to predict MetS in future. For instance, national surveys with a large population or research with limited budget can employ conventional anthropometric obesity indices in order to predict MetS in a quick manner as well as to minimise the overall costs. On the other hand, clinical practitioner or studies with lesser population and with sufficient funding can proceed with novel anthropometric obesity indices to predict MetS. Hence, the first objective of the present study aimed to compare the abilities of conventional anthropometric obesity indices (BMI, BF%, WHtR) and novel anthropometric obesity indices (LAP, VAI, ABSI and BRI) to predict MetS among Malaysian vegetarians.

To the best of our knowledge, there were noticeable differences in the body compositions between vegetarians and the general population, whereby vegetarians had lower body weight, BMI, WC, fat-free mass, BF%, adiposity and obesity than non-vegetarians^40,50,51^. Besides, vegetarians also reported with healthy lipid profile than non-vegetarians^52^. However, the optimal cut-offs to identify MetS from previous studies were established without considering the dietary patterns of the studied populations^15–17^. Notably, epidemiological studies have indicated that the majority of the vegetarians were women, attained higher educational level and higher economic status, non-smokers and non-alcohol drinkers than non-vegetarians^53,54^. Considering non-similarities between vegetarians and non-vegetarians as well as the significant associations between socio-demographic status, lifestyle factors and MetS, the universal optimal cut-offs that derived from the general population may not be applicable to predict MetS among vegetarian population. At the same time, literatures have reported differential risk of MetS, body compositions and lipid profiles between sexes^17,36^, which highlights the importance to establish sex-specific optimal cut-offs to predict MetS. In response to this, the second objective of the present study aimed to determine the sex-specific optimal cut-offs to predict MetS, particularly among Malaysian vegetarians.

## Results

### General and clinical characteristics of the vegetarians

Table 1 shows the general and clinical characteristics of the vegetarians. Both male and female vegetarians with MetS had higher value of body weight, WC, BMI, BF%, WHtR, LAP, VAI, ABSI, SBP, DBP, FBG, FBG, TG than those without MetS (*p* < 0.05). Of those vegetarians with MetS, the reported age for male (minimum age: 18 and maximum age: 74) and female (minimum age: 37 and maximum age: 67) were different. For vegetarians without MetS, the minimum age for male and female was 18, whereas the maximum age for male and female were 72 and 78, respectively. The overall prevalence of MetS among Malaysian vegetarians was 24.2% (male: 29.2%; female: 21.5%).

**Table 1:** Characteristics of the vegetarians (*n* = 273).

Variables	Male (*n* = 96)			Female (*n* = 177)		
	Non-MetS (*n* = 68)	MetS (*n* = 28)	*p* value	Non-MetS (*n* = 139)	MetS (*n* = 38)	*p* value
Age	44.6 ± 14.7	49.3 ± 13.5	0.150	47.3 ± 13.0	52.4 ± 7.9	0.003*
Age groups^a^			0.374			0.065
18–39	23 (76.7)	7 (23.3)		34 (94.4)	2 (5.6)	
40–49	19 (76.0)	6 (24.0)		41 (77.4)	12 (22.6)	
50–59	13 (56.5)	10 (43.5)		41 (73.2)	15 (26.8)	
≥ 60	13 (72.2)	5 (27.8)		23 (71.9)	9 (28.1)	
**Ethnicity**			0.054			0.007*
Chinese	34 (81.0)	8 (19.0)		92 (85.2)	16 (14.8)	
Indians	34 (63.0)	20 (37.0)		47 (68.1)	22 (31.9)	
**Smoking behaviour**^**b**^						0.518
Non-smoker	61 (70.9)	25 (29.1)	0.981	137 (78.7)	37 (21.3)	
Past smoker	7 (70.0)	3 (30.0)		2 (66.7)	1 (33.3)	
**Alcohol consumption**^**b**^			0.717			0.579
Yes	6 (66.7)	3 (33.3)		128 (78.0)	36 (22.0)	
No	62 (71.3)	25 (28.7)		11 (84.6)	2 (15.4)	
**Physical activity level**			0.431			0.072
Insufficient physical activity	24 (64.9)	13 (35.1)		76 (85.4)	13 (14.6)	
Moderately active	26 (78.8)	7 (21.2)		46 (73.0)	17 (27.0)	
Highly active	18 (69.2)	8 (30.8)		17 (68.0)	8 (32.0)	
Body weight (kg)	68.7 ± 11.3	78.9 ± 12.5	0.0001*	54.7 ± 9.0	66.7 ± 11.7	0.0001*
Height (cm)	170.6 ± 7.0	169.2 ± 6.3	0.346	157.3 ± 6.3	157.1 ± 6.0	0.825
WC (cm)	85.6 ± 9.8	98.8 ± 10.7	0.0001*	75.6 ± 8.5	88.7 ± 9.0	0.0001*
BMI (kg/m^2^)	23.7 ± 4.1	27.7 ± 3.8	0.0001*	22.1 ± 3.2	26.9 ± 3.7	0.0001*
BF (%)	24.5 ± 6.6	29.8 ± 4.2	0.0001*	30.9 ± 5.8	36.9 ± 4.7	0.0001*
WHtR	0.503 ± 0.063	0.584 ± 0.061	0.0001*	0.481 ± 0.562	0.565 ± 0.054	0.0001*
LAP	26.780 ± 16.650	75.3 ± 35.1	0.0001*	17.382 ± 11.192	63.529 ± 52.647	0.0001*
VAI	1.501 ± 1.038	3.222 ± 1.537	0.0001*	1.369 ± 0.825	3.789 ± 3.479	0.0001*
ABSI	0.799 ± 0.557	0.832 ± 0.534	0.011*	0.769 ± 0.049	0.790 ± 0.042	0.017*
BRI	2.123 ± 0.302	2.177 ± 0.260	0.405	2.727 ± 0.334	2.737 ± 0.294	0.874
SBP (mmHg)	128.6 ± 16.4	139.5 ± 13.3	0.002*	123.0 ± 19.2	136.3 ± 15.7	0.0001*
DBP (mmHg)	77.1 ± 11.1	84.0 ± 9.1	0.004*	72.0 ± 9.1	81.7 ± 10.4	0.0001*
FBG (mmol/L)	4.9 ± 0.9	6.2 ± 2.5	0.0001*	4.7 ± 0.6	5.9 ± 1.6	0.0001*
TC (mmol/L)	5.0 ± 1.1	5.5 ± 0.8	0.047*	4.7 ± 0.8	5.3 ± 1.0	0.001*
TG (mmol/L)	1.3 ± 0.9	2.3 ± 1.0	0.0001*	0.9 ± 0.5	2.2 ± 1.9	0.001*
LDL-c (mmol/L)	3.2 ± 1.0	3.2 ± 1.1	0.899	2.9 ± 0.7	3.1 ± 1.1	0.334
HDL-c (mmol/L)	1.2 ± 0.2	1.0 ± 0.2	0.0001*	1.4 ± 0.2	1.1 ± 0.2	0.0001*
**OW/OB**			0.0001*			0.0001*
Yes	20 (50.0)	20 (50.0)		29 (50.9)	28 (49.1)	
No	48 (85.7)	8 (14.3)		110 (91.7)	10 (8.3)	
**Large WC**			0.0001*			0.0001*
Yes	18 (43.9)	23 (56.1)		42 (53.8)	36 (46.2)	
No	50 (90.9)	5 (9.1)		97 (98.0)	2 (2.0)	
**High BP**			0.001*			0.0001*
Yes	34 (58.6)	24 (41.4)		47 (62.7)	28 (37.3)	
No	34 (89.5)	4 (10.5)		92 (90.2)	10 (9.8)	
**High FBG**			0.0001*			0.0001*
Yes	8 (35.4)	14 (63.6)		9 (32.1)	19 (67.9)	
No	60 (81.1)	14 (18.9)		130 (87.2)	19 (12.8)	
**High TG**			0.0001*			0.0001*
Yes	13 (36.1)	23 (63.9)		11 (35.5)	20 (64.5)	
No	55 (91.7)	5 (17.9)		128 (87.7)	18 (12.3)	
**Low HDL-c**			0.0001*			0.0001*
Yes	5 (27.8)	13 (72.2)		37 (55.2)	30 (44.8)	
No	63 (80.8)	15 (19.2)		102 (92.7)	8 (7.3)	

Variables are presented as Mean ± SD and n (%).

*MetS* metabolic syndrome, *WC* waist circumference, *BMI* body mass index, *BF%* body fat percentage, *WHtR* waist-to-height ratio, *LAP* lipid accumulation product, *VAI* visceral adiposity index, *ABSI* a body shape index, *BRI* body roundness index, *SBP* systolic blood pressure, *DBP* diastolic blood pressure, *FBG* fasting blood glucose, *TC* total cholesterol, *TG* triglyceride, *LDL-c* low-density lipoprotein cholesterol, *HDL-c* high-density lipoprotein cholesterol, *OW* overweight, *OB* obesity.

*Indicates a significant difference at *p* < 0.05 by Chi-square test or Independent samples t-test.

^a^Age groups were merged into three categories to perform valid Chi-square analysis, with value reported in ${\upchi }$^2^ and *p*.

^b^Variables were tested using Fisher Exact test due to the cells had expected count of less than 5.

### Abilities of the conventional and novel anthropometric obesity indices to predict MetS among male vegetarians

As depicted in Table 2, all conventional anthropometric obesity indices have the abilities to distinguish MetS case from the non-MetS case (*p* < 0.05). The AUCs to predict MetS according to the BMI, BF%, WHtR were 0.777, 0.782, 0.825, respectively. Of those conventional anthropometric obesity indices, WHtR had largest AUC (0.825, *p* = 0.0001) than BMI (AUC: 0.777, *p* = 0.0001) and BF% (AUC: 0.782, *p* = 0.0001). However, the AUC of WHtR was not statistically different than BMI and BF% when we further tested the AUCs’ using paired design ROC curve analysis provided in NCSS statistical software. Despite so, WHtR remained as a proposed index to determine the MetS in male vegetarians due to its high PPV as compared to BMI and BF%. The proposed optimal cut-off value based on the WHtR to predict MetS among male vegetarians was 0.571.

**Table 2:** AUCs, optimal cut-off, sensitivity, specificity for obesity indices based on the ROC curve analysis in identifying MetS and its components among male vegetarians (*n* = 96).

	AUC (95% CI)	*p* value^a^	YI	Cut-off	Sn (%)	Sp (%)	PPV	NPV	*p* value^b^	*p* value^c^
**Conventional index**										
BMI (kg/m^2^)	0.777 (0.686–0.868)	0.0001*	0.471	23.000	1.000	0.471	0.438	1.000	0.168	–
BF%	0.782 (0.685–0.878)	0.0001*	0.536	28.050	0.786	0.750	0.564	0.895	0.361	–
WHtR	0.825 (0.739–0.910)	0.0001*	0.571	0.541	0.821	0.750	0.575	0.911	–	–
**Novel index**										
LAP	0.923 (0.867–0.980)	0.029*	0.710	41.435	0.857	0.853	0.706	0.935	–	–
VAI	0.864 (0.786–0.942)	0.040*	0.660	2.231	0.821	0.838	0.677	0.919	–	0.044*
ABSI	0.652 (0.534–0.770)	0.060	0.252	0.780	0.679	0.574	0.396	0.812	–	0.0001*
BRI	0.583 (0.457–0.709)	0.064	0.208	2.248	0.429	0.779	0.445	0.768	–	0.0001*

*BMI* body mass index, *BF%* body fat percentage, *WHtR* waist-to-height ratio, *LAP* lipid accumulation product, *VAI* visceral adiposity index, *ABSI* a body shape index, *BRI* body roundness index, *AUC* area under the curve, *95% CI* 95% confidence interval, *YI* Youden’s Index, *Sn* sensitivity, *Sp* specificity, *PPV* positive predictive value, *NPV* negative predictive value.

*Indicates a significant difference at *p* < 0.05 by ROC analysis.

^a^Ability of each index to separate MetS and non-MetS.

^b^Comparison of the AUC value of WHtR with BMI and BF%.

^c^Comparison of the AUC value of LAP with VAI, ABSI and BRI.

With regards to novel anthropometric obesity indices, all novel anthropometric obesity indices had the ability to predict the MetS, except for ABSI (AUC: 0.652, *p* = 0.060) and BRI (AUC: 0.583, *p* = 0.064). Within the novel anthropometric obesity indices, LAP (AUC: 0.923, *p* = 0.029) was identified as a better predictor to identify MetS due to its greater AUC than VAI (AUC: 0.880, *p* = 0.0001). LAP remained as a proposed index to determine the MetS in male vegetarians due to its high Sn, SP, NPV and PPV than VAI. Based on the ROC curve analysis, the proposed optimal cut-off value of LAP to predict MetS was 41.435 among male vegetarians. While the LAP and VAI had successfully distinguished the MetS case from the non-MetS case, the ROC curve analysis found that ABSI and BRI were not effective to predict MetS (*p* > 0.05).

**Figure 1:**
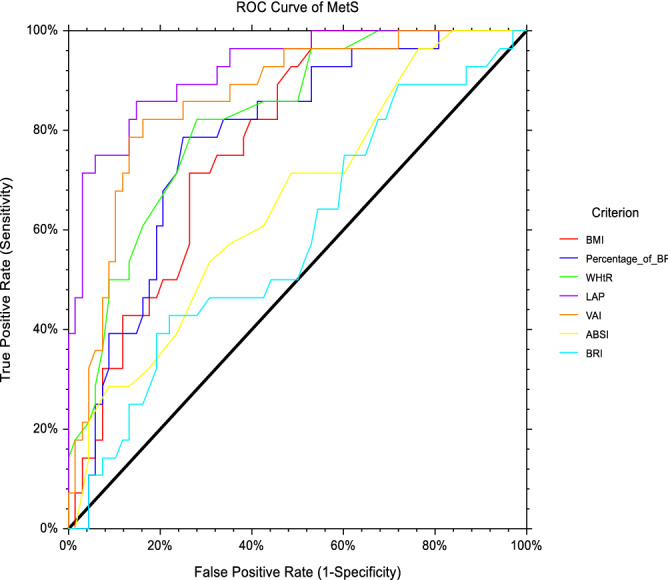
ROC curve analysis of anthropometric obesity indices to predict MetS among male vegetarians. *BMI* body mass index, *BF%* body fat percentage, *WHtR* waist-to-height ratio, *LAP* lipid accumulation product, *VAI* visceral adiposity index, *ABSI* a body shape index, *BRI* body roundness index, *ROC curve* receiver operating characteristic curve, *MetS* metabolic syndrome.

The ROC curve analysis in Fig. 1 shows the abilities of different anthropometric indices to predict MetS among male vegetarians in Malaysia.

### Abilities of the conventional and novel anthropometric obesity indices to predict MetS among female vegetarians

Table 3 shows that all conventional anthropometric obesity indices have the abilities to distinguish MetS case from the non-MetS case (*p* < 0.05). As presented in Table 3, WHtR (AUC: 0.863, *p* = 0.0001) had a greater ability to predict the MetS as compared to BMI (AUC: 0.847, *p* = 0.0001) and BF% (AUC: 0.785, *p* = 0.0001) among women vegetarians in Malaysia. However, the AUC of WHtR was not statistically different from BMI when being further tested their AUCs’ differences using paired design ROC curve analysis. The suggested WHtR’s optimal cut-off value to predict the MetS was 0.532.

**Table 3:** AUCs, optimal cut-off, sensitivity, specificity for obesity indices based on the ROC curve analysis in identifying MetS among female vegetarians (*n* = 177).

	AUC (95% CI)	*p* value^a^	YI	Cut-off	Sn (%)	Sp (%)	PPV	NPV	*p* value^b^	*p* value^c^
**Conventional index**										
BMI (kg/m^2^)	0.847 (0.782–0.913)	0.0001*	0.576	24.050	0.842	0.734	0.464	0.944	0.503	–
BF%	0.785 (0.708–0.863)	0.0001*	0.437	34.500	0.711	0.727	0.416	0.902	0.006*	–
WHtR	0.863 (0.797–0.928)	0.0001*	0.614	0.532	0.816	0.799	0.526	0.941	–	–
**Novel index**										
LAP	0.920 (0.862–0.977)	0.0001*	0.652	21.743	0.947	0.705	0.468	0.980	–	–
VAI	0.882 (0.818–0.947)	0.0001*	0.713	1.965	0.842	0.871	0.640	0.953	–	0.143
ABSI	0.636 (0.536–0.735)	0.010*	0.250	0.777	0.632	0.619	0.312	0.860	–	0.0001*
BRI	0.537 (0.436–0.639)	0.480	0.154	2.714	0.579	0.576	0.272	0.833	–	0.0001*

*BMI* body mass index, *BF%* body fat percentage, *WHtR* waist-to-height ratio, *LAP* lipid accumulation product, *VAI* visceral adiposity index, *ABSI* a body shape index, *BRI* body roundness index, *AUC* area under the curve, *95% CI* 95% confidence interval, *YI* Youden’s Index, *Sn* sensitivity, *Sp* specificity, *PPV* positive predictive value, *NPV* negative predictive value.

*Indicates a significant difference at *p* < 0.05 by ROC analysis.

^a^Ability of each index to separate MetS and non-MetS.

^b^Comparison of the AUC value of WHtR with BMI and BF%

^c^Comparison of the AUC value of LAP with VAI, ABSI and BRI.

With regards to novel anthropometric obesity indices, all novel anthropometric obesity indices had the ability to predict the MetS, except for BRI (AUC: 0.537, *p* = 0.480). The present study found that LAP (AUC: 0.920, *p* = 0.0001) had a better ability to predict the MetS than VAI (AUC: 0.882, *p* = 0.0001), ABSI (AUC: 0.636, *p* = 0.010) and BRI (AUC: 0.537, *p* = 0.480). Based on the ROC curve analyses, the suggested LAP’s optimal cut-off value to predict the MetS was 21.743.

**Figure 2:**
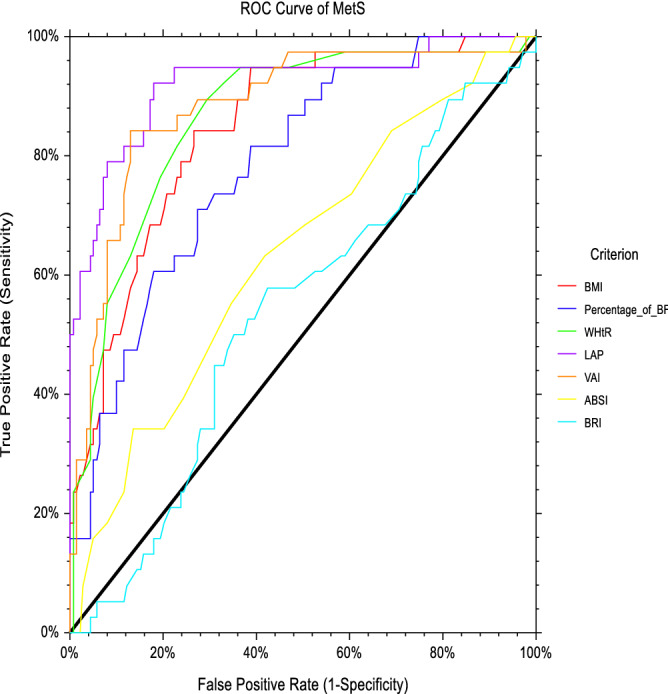
ROC curve analysis of anthropometric obesity indices to predict MetS among female vegetarians. *BMI* body mass index, *BF%* body fat percentage, *WHtR* waist-to-height ratio, *LAP* lipid accumulation product, *VAI* visceral adiposity index, *ABSI* a body shape index, *BRI* body roundness index, *ROC curve* receiver operating characteristic curve, *MetS* metabolic syndrome.

The ROC curve analysis in Fig. 2 shows the abilities of different anthropometric indices to predict MetS among female vegetarians in Malaysia.

Overall, the present study found that WHtR was the best index in conventional category and LAP was the best index in novel category to predict MetS. Table 4 shows the comparison between the ability of WHtR and LAP to predict MetS in both sexes. Further comparison using paired design ROC curve analysis found that the AUC of LAP (AUC: 0.923) was significantly larger than WHtR (AUC: 0.825, *p* = 0.016) among male vegetarians. For female vegetarians, the AUC of LAP (AUC: 0.920, *p* = 0.0001) was larger than WHtR (AUC: 0.863, *p* = 0.0001) to predict the MetS (*p* = 0.012).

**Table 4:** Further comparison between the ability of WHtR and LAP to predict MetS in both sexes.

	WHtR’s AUC	LAP’s AUC	WHtR’s AUC–LAP’s AUC	Difference (%)	*p* value
Male (n = 96)	0.825	0.923	− 0.098	11.9	0.016*
Female (n = 177)	0.863	0.920	− 0.057	6.6	0.012**

*WHtR* waist-to-height ratio, *LAP* lipid accumulation product, *AUC* area under the curve, *95% CI* 95% confidence interval, *YI* Youden’s Index, *Sn* sensitivity, *Sp* specificity, *PPV* positive predictive value, *NPV* negative predictive value.

*Indicates a significant difference at *p* < 0.05 by ROC analysis.

## Discussion

The double burden of obesity and MetS has become an emerging health threat in the worldwide population. The prevailing of both obesity and MetS highlight the importance to identify a suitable tool to discriminate the MetS at an early stage. Though there were numerous studies have determined the ability of anthropometric obesity indices to predict the MetS, none of the proposed obesity indices was made for the vegetarian population^55–57^. To the best of our knowledge, the findings of the present study are new as the present study is the first study to compare the abilities of seven different anthropometric obesity indices in predicting MetS. Additionally, the proposed sex-specific optimal cut-offs to predict the MetS may serve as a useful reference for the health personnel to identify the MetS among those on a vegetarian diet in Malaysia in the future.

The overall prevalence of MetS in the present study (24.2%) was lower than the general population in Malaysia (42.5%)^8^. When we stratified the findings based on sex, the prevalence of MetS among men and women vegetarians were 29.2% and 21.5%, respectively, which are lower than the general population in Malaysia^8,58^. The overall prevalence of abdominal obesity (44.1%) among female vegetarians is lower as compared to the general women population in Malaysia (60.2%)^59^. On the other hand, there were a greater number of male vegetarians (42.7%) were abdominal obese as compared to the general population (38.2%) in Malaysia. A higher number of women vegetarians were obese (32.2%) based on BMI than the general women population (20.6%) in Malaysia^59^, and a higher number of vegetarians being classified as obese by using WC (male: 42.7%; female: 44.1%) than BMI (male: 41.7%; female: 32.2%). These findings highlight the possibilities of misclassification of the obese individual into healthy body weight status by using BMI. Hence, the present optimal cut-offs values that were derived from the general population might not be applicable to the vegetarian population due to the differential risk of obesity were observed.

When we separated the anthropometric obesity indices into the conventional and novel category, we found that all conventional anthropometric obesity indices (BMI, BF% and WHtR) were useful to predict MetS in both sexes. Of those conventional anthropometric obesity indices, WHtR was the best predictor to identify the MetS due to its largest AUCs within conventional anthropometric obesity index category. These findings are in accord with the previous studies in Caucasians and Asians population^15,60,61^. The better ability of WHtR to predict MetS than BMI and BF% may due to the ability of WHtR to differentiate the distribution of adipose tissues, which play a significant role than total body fat percentage in the development of MetS^17^. Additionally, WHtR is relatively stable to act as an “early health risk” as it considers the height of an individual^57^. Secondly, WHtR could identify an individual with higher metabolic risk among individual with a moderate range of BMI due to the close association between WHtR and central obesity than BMI^21^. In contrast to BMI and BF%, WHtR take the individual height into consideration in assessing obesity. It is important to consider an individual height as some studies have shown that shorter individuals had a higher risk of getting CVD, ischemic heart disease, T2DM and premature death^62,63^. Due to the drawbacks of BMI and BF%, WHtR appeared as a better index to predict MetS within the conventional anthropometric obesity index category.

On the other hand, the present study found that LAP and followed by VAI were useful novel anthropometric obesity indices in predicting the MetS among vegetarians in both sexes. The ability of LAP to predict MetS was documented in Taiwan older adults and China adult’s population^30,47^. The use of LAP offers more advantages to predict MetS as it reflects the visceral adiposity level in the human body. Visceral adipose tissue metabolism exerts an impact on whole-body metabolism, whereby it produces more interleukin-6 (IL-6) and plasminogen activator inhibitor-1 (PAI-1) than subcutaneous adipose tissue^64^. Furthermore, Chiang and Koo^30^ found that the integration of TG in LAP formula was able to identify individuals with a higher amount of visceral fat. Considering individuals with MetS often reported with higher IL-6 and PAI-1 than the non-MetS individual as well as a significant association between TG and visceral adipose tissues, it is crucial to have a routine check-up of visceral adiposity using LAP assessment. VAI ranked in the second position to predict MetS among Malaysian vegetarians, which is in concordance with previous studies^47,65^. Furthermore, the calculation of VAI is more complicated than LAP as it involves the values of WC, BMI, TG and HDL-c. It is recommended to use LAP instead of VAI to predict MetS.

In the present study, we found that ABSI was useful to predict MetS in female, but not in male vegetarians. Previous studies conducted in Spain concluded that ABSI should not be used to predict MetS^37^ and insulin resistance in China^35^. The mean difference in the individual height could impact the ability of ABSI to determine the MetS^37^. Hence, it’s critical to take the mean individuals’ height into consideration wherever using the ABSI method. Another thing to be highlighted is ABSI is derived based on the American population^32^. Since Asians were at greater risk of abdominal obesity and low muscle mass than Caucasians, it is therefore the coefficients of ABSI that derived from American population might not be applicable to other population, especially for Asian populations^35^. On the other hand, BRI was the poor index to identify MetS in both sexes, which is similar to the previous research in Poland^17^. Despite so, another study found that BRI was useful to predict the risk of MetS in both sexes among Peruvian population^36^. In response to this, BRI could hardly be applied to predict MetS for both sexes in the local context, therefore, future studies can consider modifying the BRI for better application among the Malaysian population. Besides, the different predicting performance of these studies could be attributed to the variation in MetS criteria, and respondents’ characteristics^37^.

Considering the differential risk of MetS were observed in previous studies^17,36^, specific optimal cut-offs to predict MetS were formed for WHtR and LAP in the present study. The optimal cut-off for WHtR to predict MetS for male vegetarians and female vegetarians were 0.541 and 0.532, respectively, which are higher than the general population in China (male: 0.510; female: 0.510)^66^ and South Indian (male: 0.520; female: 0.506)^60^. These data suggest that the application of WHtR’s optimal cut-off which derived from the general population may underestimate the MetS among the vegetarian population in Malaysia. Besides, the present recommended WHtR value for MetS among vegetarians in Malaysia is also higher than the general statement of ‘keep your waist to less than half your height’ or WHtR of 0.50*.* The present result indicates that the WHtR less than 0.50 may not be appropriate to identify MetS for vegetarians in Malaysia. A plausible explanation to explain the result disparities is due to a different degree of adiposity between vegetarians and non-vegetarians, whereby non-vegetarians had a higher body fat percentage than vegetarians^50,67^. Therefore, a higher optimal cut-off WHtR value is needed among the vegetarian population.

On the other hand, the proposed optimal cut-offs to predict the MetS based on the LAP were 41.435 for male and 21.743 for female vegetarians. There are remarkable big differences in the proposed LAP’s optimal cut-offs for MetS as compared to China^47^ and Turkey^65^. The proposed optimal cut-offs to predict the MetS for men and women were 27.29 and 36.45 as reported among hemodialysis patients in China^47^. Likewise, another study that was conducted among the Turkish population with kidney disease reported the optimal cut-offs to predict the MetS for men and women were 36.60 and 33.50, respectively. The differences in optimal cut-offs for MetS from the previous studies could be attributed to respondents’ characteristics. Notably, both respondents from former studies^47,65^ were patients with stage 3 to stage 5 chronic kidney disease or hemodialysis patients. Up to-date, to the best of our knowledge, there is one published data on the optimal cut-off to predict MetS among the healthy generation population in Iran^68^. The proposed optimal cut-off values to predict MetS in for male and female in Iran were 39.89 and 49.71, respectively. Though the former study^68^ was conducted among the healthy population, the respondents’ dietary patterns were not presented in their study, which makes the direct comparison become infeasible. Furthermore, the divergent optimal cut-off values may be due to anthropometric and racial differences^65,68^. Since this is the first study to develop optimum sex-specific optimal cut-offs for MetS vegetarians, more local studies are needed in the future.

Overall, the present study found that WHtR and LAP were best indices to predict the MetS when we sought the findings based on the conventional and novel category. The further comparison between the WHtR and LAP found that the AUC of LAP was significantly larger than WHtR, which are similar to the past studies^30,47,65^. The plausible reason to explain the better ability of LAP than WHtR to predict MetS could be probably due to the additional input of TG in LAP. LAP covers two of the MetS components, namely WC and LAP, whereas WHtR covers one of the MetS components, which is WC. As the presence of MetS is diagnosed according to the number of MetS components, therefore, the additional TG in LAP increases its chance to predict MetS than WHtR. In conjunction with previous studies conducted among patients^47,65^, the present findings support the usefulness of LAP for the identification of MetS across different ethnic groups and different diseases. Despite the superior predictability of LAP for identification of MetS, LAP might not be an ideal index to predict MetS in a large community project that require instant checking of MetS status. It would be useful if a simpler index is available for easy diagnosis of individuals at risk of MetS in large epidemiology survey or clinical settings. Thus, WHtR that derived from WC and height of an individual can be used as an alternative to replace LAP to identify MetS whenever instant checking of MetS is required.

The present study has some limitations. Firstly, the present study is a cross-sectional study, which is unable to detect the cause-effect association of obesity indices and MetS. Prospective studies are recommended to affirm the association of obesity indices with MetS in the future. Besides, the current application of the YI is limited to two diagnostic groups (non-disease and disease). Nevertheless, most of the diseases have the transitional intermediate stage (eg: non-hypertension, prehypertension and hypertension). The drawback of YI to identify the intermediate stage of disease may lead to delay in providing necessary intervention for those in the transitional intermediate stage of the diseases^69^. In contrast, early recognition of the intermediate stage of diseases may prevent the progression of the disease to severe disease stage. Next, the present study focuses among vegetarians and the abilities of anthropometric obesity indices to predict MetS remains unknown among the population with different dietary patterns such as non-vegetarian population in Malaysia. Considering the significant associations between dietary patterns and MetS^70^, future study should further compare the abilities of anthropometric obesity indices to predict MetS in order to cover more population with different dietary patterns. Meanwhile, the findings of the present study are only applicable to vegetarians in Malaysia, and the proposed optimal cut-offs values might not be applicable to other overseas vegetarians due to racial differences. Considering different body compositions between ethnicity, more studies are needed to determine specific optimal cut-offs among other vegetarians, especially vegetarians from Western countries. Another limitation is our study did not stratify the vegetarians according to their ethnicity based on sexes due to sample size limitation. Malaysia is a multi-ethnic country consists of Malay, Chinese and Indians, whereby Chinese and Indians are two main ethnicities practising vegetarianism in Malaysia. Notable differences in body compositions between the ethnicities^71^, which could be potential confounder to affect the associations between anthropometric obesity indices and MetS. Therefore, future studies are needed to determine sex-specific cut-offs based on ethnicity, particular among vegetarian population in Malaysia. Despite the limitations, the present study is the first vegetarian study to compare the abilities of conventional anthropometric obesity indices and novel anthropometric obesity indices to predict MetS, which provide more selections for the future researchers and health care professionals. Besides, the present study also the first study to determine the sex-specific optimal cut-off points for MetS among vegetarian population, which help to diagnose MetS at an early stage.

## Conclusion

In conclusion, LAP was the best indicator to predict the MetS. However, WHtR could be an alternative anthropometric obesity index in large epidemiology survey due to its convenient and cost-effective characteristics. Our study suggests that future studies with sufficient funding can use LAP to predict MetS, whereas future studies with a large population or limited budget can use WHtR for the prediction of MetS. In the present study, the suggested WHtR’s optimal cut-offs and LAP’s optimal cut-offs for MetS for male and female vegetarians were 0.541, 0.532, 41.435 and 21.743, respectively. Health professionals can utilise the current optimal cut-offs for identification of MetS among vegetarian in Malaysia in the future.

## Methods

### Study population

The ethical approval for the study was obtained from the Ethics Committee for Research involving Human Subjects, Universiti Putra Malaysia (JKEUPM), with a reference number of FPSK (FR16) P023. The methods in this study were carried out in accordance with relevant guidelines and regulations that comply with institutional, national, or international guidelines. Details of the study had been published elsewhere^41,72^. The sample size of the present study was calculated using the proportion formula^73^ and the prevalence of MetS among vegetarians (13.0%) as depicted in the previous study^13^. With 80% statistical study power^73^, 1.1 of design effect^74^ and non-response rate as shown in previous research (30.0%)^58^, a total of 273 vegetarians were required for the present study.

The present study was conducted among the selected religious community centres (Buddhist and Hindu) in Kuala Lumpur and Selangor, Malaysia. In Malaysia, Buddhist and Hindu are two common groups who adopt vegetarianism. Meanwhile, the selected religious community centres are common places for the vegetarians to participate in religious activities. Simple random sampling was used to select a total of nine religious’ community centres from 31 religious community centres based on the lists of community centres provided by headquarters. After the selection of religious centres, cluster sampling was used to invite all members of the selected community centres who fulfilled the following study criteria, namely, adults aged above 18 years old, practising vegetarianism for more than two years, not pregnant or lactating, and not taking medications in controlling dyslipidaemia, diabetes mellitus, and hypertension to participate in the present study. Prior to data collection, the purposes and protocol of the study were explained to members of the selected community centres using a study information sheet. Informed consents have been obtained from the study respondents and they were requested to fast overnight before the data collection day. A total of 355 respondents consented to participate in the study before the day of the data collection. However, 82 of them were excluded from the study due to absenteeism, did not fast for the blood withdrawal or failure to fulfil the inclusion and exclusion criteria of the study. Hence, a total of 273 respondents who fulfilled the study criteria were included in the present study.

### Self-administered questionnaire

Information of respondents such as age, sex and ethnicity were self-reported by respondents during the data collection. The Global Adult Tobacco Survey (GATS) was used to determine the smoking behaviour of respondents^75^. Respondents were classified into past smokers, current smokers and non-smokers. Alcohol consumption of respondents was determined by the adapted alcoholic questionnaire taken from the National Health and Nutrition Examination Survey (NHANES) Food Frequency Questionnaire (FFQ)^76^. Respondents were classified into alcohol drinkers and non-alcohol drinkers. The Global Physical Activity Questionnaire (GPAQ) was used to determine the physical activity level of the respondents. Respondents were classified into three categories, namely insufficient physical activity, moderately active and highly active^77^.

### Anthropometric and blood pressure measurements

Anthropometric measurements were done according to the standard protocol in the International Society for the Advancement of Kinanthropometry (ISAK) method^78^. Height of the respondents was measured in centimetre (cm) using a SECA213 portable stadiometer (SECA, Hamburg, Germany) to the nearest of 0.1 cm. Respondents were requested to stand bare feet with their head placed in the Frankfort plane by not touching the scale. Respondents were requested to take and hold a deep breath while keeping their head in the Frankfort plane for the height measurements. Afterwards, the recorder was placed on the head of the respondents by the researcher. The recorder needs to be compressed the respondents’ hairs as much as possible. The measurement of the height was taken by the researcher while the respondents holding their breath. Body weight of the respondents was measured in kilogram (kg) using TANITA Digital Weight Scale HD306 (TANITA Corporation, USA) to the nearest 0.1 kg. Respondents were asked to remove objects from their pocket and stand bare feet on the weighing scale. Body mass index (BMI) of the respondents was calculated as kg/m^2^ and classified into four categories according to the World Health Organization, namely underweight (< 18.5 kg/m^2^), normal weight (18.5–24.9 kg/m^2^), overweight (25.0–29.9 kg/m^2^) and obesity (≥ 30 kg/m^2^)^79^. WC of the respondents was measured in centimetre (cm) using a Lufkin tape W606PM (Lufkin, USA) to the nearest of 0.1 cm. Respondents were asked to stand with feet closed together with arms at the side. Respondents were asked to breathe normally and the measurements of WC were taken at the mid-point between the lower costal border and the iliac crest during the end of the expiration by the researcher. Body fat percentage (BF%) was measured using the Omron body fat analyser HBF-306-E (Omron Corporation, Japan). Information such as age, sex, body weight and height of the respondent were entered into the body fat analyser by the researcher. During the measurement of BF%, respondents were asked to hold their arms at a 90-degree angle and placed their hands on the grips of the body fat analyser. Next, systolic blood pressure (SBP) and diastolic blood pressure (DBP) of the respondents were measured using an Omron automatic blood pressure monitor HEM-7121 (Omron Corporation, Japan). Respondents were requested to rest in a sitting position for at least 5 min before measurement. The first measurement of BP was measured at the right arm of the respondents. The second measurement of blood pressure was taken after another 5 min of resting interval. The BP value was calculated based on the average value of the BP’s measurements.

### Calculation of anthropometric indices

Conventional anthropometric indices (BMI and WHtR) and novel anthropometric obesity indices (LAP, VAI, ABSI and BRI) were calculated based on the following formulas:

$$ {\text{BMI }}\,\left( {{\text{kg}}/{\text{m}}^{{2}} } \right) \, = \frac{{{\text{Body weight}} \,\left( {{\text{kg}}} \right)}}{{{\text{Height}} \,\left( {\text{m}} \right)^{2} }} $$

$$ {\text{WHtR }} = \frac{{{\text{WC}}\,\left( {{\text{cm}}} \right)}}{{{\text{Height}} \,\left( {{\text{cm}}} \right)}} $$

$$ {\text{LAP}}_{{{\text{male}}}} = \left[ {{\text{WC}} \,\left( {{\text{cm}}} \right) - 65} \right] \times {\text{TG}} \,\left( {\text{mmol/L}} \right)^{49} $$

$$ {\text{LAP}}_{{{\text{female}}}} = \left[ {{\text{WC}} \,\left( {{\text{cm}}} \right) - 58} \right] \times {\text{TG}} \,\left( {\text{mmol/L}} \right)^{49} $$

$$ {\text{VAI}}_{{{\text{male}}}} = \left[ {\frac{{{\text{WC}} \,\left( {{\text{cm}}} \right)}}{{39.68 + 1.88 \times {\text{BMI}}\,\left( {\frac{{{\text{kg}}}}{{{\text{m}}^{2} }}} \right)}}} \right] \times \left[ {\frac{{{\text{TG}} \,\left( {\text{mmol/L}} \right)}}{1.03}} \right] \times \left[ {\frac{1.31}{{{\text{HDL}} \,\left( {\text{mmol/L}} \right)}}} \right]^{31} $$

$$ {\text{VAI}}_{{{\text{female}}}} = \left[ {\frac{{{\text{WC}} \,\left( {{\text{cm}}} \right)}}{{36.58 + 1.89 \times {\text{BMI}} \,\left( {\frac{{{\text{kg}}}}{{{\text{m}}^{2} }}} \right)}}} \right] \times \left[ {\frac{{{\text{TG}} \,\left( {\text{mmol/L}} \right)}}{0.81}} \right] \times \left[ {\frac{1.52}{{{\text{HDL}} \,\left( {\text{mmol/L}} \right)}}} \right]^{31} $$

$$ {\text{ABSI }} = \frac{{{\text{WC}} \,\left( {\text{m}} \right)}}{{{\text{BMI}} \,\left( {{\text{kg/m}}^{2} } \right) ^{2/3} \times {\text{Height}} \,\left( {\text{m}} \right)^{1/2} }}^{32} $$

$$ {\text{BRI }} = { 364}.{2}{-}{365}.{5} \times \sqrt {1 - \frac{{\,\left( {{\text{WC}}\,\left( {\text{m}} \right)/2{\uppi }} \right)^{2} }}{{[0.5 \times {\text{height}} \,\left( {\text{m}} \right)]^{2} }}}^{80} $$

### Biochemical measurements

A total of 10 ml of the overnight venous fasting blood sample was used for the fasting blood glucose (FBG) level, triglyceride (TG), high-density lipoprotein cholesterol (HDL-C) determination. All samples were analysed using an Olympus Au analyser (AU640, Beckman Olympus, Brea, CA, USA).

### Definition of MetS

According to the Joint Interim Statement (JIS) 2009, respondents were considered as having the MetS when three or more than three out of the five MetS components are present. The five MetS components including the abdominal obesity (≥ 90.0 cm for men and ≥ 80.0 cm for women according to Asian cut-offs), high BP (SBP ≥ 130 mmHg or DBP ≥ 85 mmHg), high FBG (≥ 5.6 mmol/L), high TG (≥ 1.7 mmol/L), and low HDL-c (< 1.0 mmol/L for men and < 1.3 mmol/L for women)^1^.

### Statistical analyses

Statistical analyses were conducted using IBM SPSS statistic version 24.0 (Chicago, IL, USA). All variables that fall within skewness of ± 2 were considered normally distributed^81^. Continuous variables were presented as mean and standard deviation (mean ± SD) for normally distributed variables or median (interquartile range-IQR) for non-normally distributed variables. Categorical variables were presented as frequencies and percentage (n, %). The comparison of abilities of conventional anthropometric obesity indices (BMI, BF% and WHtR) and novel anthropometric obesity indices (LAP, VAI, ABSI and BRI) to predict MetS were analysed according to the receiver operating characteristic (ROC) curve analysis. ROC curve analysis was used to obtain the area under the curve (AUC), sensitivity (Sn), specificity (Sp), positive predictive value (PPV) and negative predictive value (NPV) for each obesity index. All AUCs were further compared to determine the efficacy of the various obesity indices in identifying the MetS using NCSS statistics version 20.0.3 (Utah, USA). Anthropometric obesity index with the largest AUC considered as a better diagnostic tool to predict MetS. The establishment of the sex-specific optimal cut-off points to define MetS were derived from the Youden’s index (YI). The sex-specific optimal cut-offs points are the diagnostic points to differentiate between MetS cases and non-MetS cases in the present study. The levels of significance for all analyses were set at *p* < 0.05.
